# Correlates of poor self-rated health among school-going adolescent girls in urban Varanasi, India

**DOI:** 10.1186/s12889-023-16822-1

**Published:** 2023-10-04

**Authors:** Ratna Patel, Dhananjay W. Bansod

**Affiliations:** Department of Public Health and Mortality Studies, International Institute for Population Sciences, Mumbai, India

**Keywords:** Self-rated health, Adolescent girls, Urban setting, India

## Abstract

**Background:**

The concept of self-rated health (SRH) has widely been studied among the adults and older population in developed as well as developing countries, including India. Also, studies are available in abundance examining the various concepts of SRH among adolescents. However, in India, studies on the SRH of adolescents remain scarce, especially those aiming to understand the correlates of SRH among school-going adolescent girls in an urban setting. Therefore, this study aims to determine the correlates of poor SRH among school-going adolescent girls in the urban setting of Varanasi, India.

**Methods:**

This study is based on the primary data collected in the Varanasi district of Uttar Pradesh, India, from October 2019 to February 2020. Nearly 350 adolescent girls and their mothers were personally interviewed. Self-rated health was the primary outcome variable of this study. The exact wording of the question asked from the adolescent girls was, “In general, how would you say your health is?”.

**Results:**

Almost one-fifth (19.4%) of the adolescent girls reported poor SRH. Adolescent girls from Other Backward Class (OBC) [OR: 0.39; 95% CI: .18-.85] and Others caste [OR: 0.58; 95% CI: .23–0.87] were less likely to report poor SRH than their Scheduled Caste/Scheduled Tribe (SC/ST) counterparts. Girls residing in households where number of daughters were more than sons were more likely to report poor SRH [OR: 7.8; 95% CI: 1.5–39.5] than girls who belonged to the daughters only households.

**Conclusion:**

Composition of children was one of the important factors as outlined in this study. The role of mothers in improving the overall SRH of the girls is critical as they are involved in caring process of their daughters.

**Supplementary Information:**

The online version contains supplementary material available at 10.1186/s12889-023-16822-1.

## Background

Social surveys rely heavily on self-reported data of health [1]. Social surveys are known to provide reliable estimates on health outcomes, as a result, in social surveys, a single-item question to measure the health of the population, popularly known as self-rated health (SRH), is widely used [2]. Social surveys have find its applicability in estimating the reliable health indicators including self-rated health. Even in many large social surveys, the question on SRH has found its applicability, thereby making it a widely used and most popular single-item measure of population health worldwide [3, 4]. Various studies have confirmed the reliability and validity of SRH as a measure of health across different sub-populations [4–9].

Though SRH has been noted as a reliable measure to capture the health status of the population, the reliability might vary between different population sub-groups as differences in the relationship between SRH and other health measures by gender and age have been reported previously [10, 11]. Despite the widespread applicability of SRH as a measure to determine health, some authors have augmented the concerns that different social groups may interpret the concept of health in different ways, thereby influencing the responses on health status measures [12]. Cultural and linguistic conventions of describing symptoms and health have been found to vary between ethnic groups [13], which may affect the measurement of SRH among various ethnic groups [13]. To that effect, interpretation of SRH has been circumspect while comparing different ethnic or cultural groups [13, 14].

Adolescents are more capable to provide response for their self-rated health than children as adolescents are grown up and understand their health in a better way than children do. Children view themselves in regards to their day-to-day activities, and therefore their response on SRH may depend on immediate cues in their surroundings contexts [15]. However, with the transition to adolescence, children begin to visualize themselves in more generalized terms, and adolescents’ identities begin to take on enduring aspects [16]. The need for consistency in self-concept at this stage of life is a reverberation of the rapid biological, physiological, and social changes occurring in their lives [17]. Therefore, during adolescence self-concept model may more adequately capture the meaning of SRH items among adolescents [2].

The concept of SRH has widely been studied among the adults and older population in developed [18–20] as well as developing countries [21–24], including in India [25–27]. Some studies have also drawn comparisons in SRH among older populations between developed and developing countries [28]. Also, studies are available in abundance examining the various concepts of SRH among adolescents [2, 29–34]. However, in India, studies on the SRH of adolescents remain scarce, especially those aiming to understand the correlates of SRH among school-going adolescent girls in an urban setting. Therefore, this study aims to determine the correlates of poor SRH among school-going adolescent girls in the urban setting of Varanasi, India.

### Data and methods

This study is based on the primary data collected in the Varanasi district of Uttar Pradesh, India, from October 2019 to February 2020. A total of 350 adolescent girls and their mothers were personally interviewed. Supplementary file 3 and supplementary file 4 are the structured schedule used for the interview.

#### Sample size estimation

The sample size may not be true representative for calculating mean SRH in this population as this study is a part of PhD research work where the focus was to explore educational outcomes, health outcomes (SRH), educational achievements and aspirations through the lens of social capital among adolescent girls. Since, the focus was to understand several outcomes including education and health, and it was difficult to reach a sample size that could serve the purpose for each objectives. However, one thing was common for each of the four objectives in the researcher’s PhD work and that was the sample included school-going girls. Therefore, we have taken the proportion of literate girls to reach our sample size.

Furthermore, it may not be justifiable to equate literacy with the school-going. However, according to the Census of India, “person aged seven and above, who can both read and write with understanding in any language, is treated as literate.” We can assume that girls in our sample already had primary (up to fifth standard, aged approximately 10–11 years) and a part of secondary education (up to Eighth standard, aged approximately 11–13 years) and therefore we can call them as literate as they know to write and read in any one of the prescribed language. Therefore, we have taken literate girls in the age group 13–19 years to reach our sampling size.

The study was conducted on school-going girls (8^th^ standard to 12^th^ standard) between 13–19 years of age.

For taking prevalence, the number of literate girls in the urban area of Varanasi, as per census 2011, in the age group 13–19 is taken as the numerator, and total girls in the age group, 13–19, are taken as the denominator.

$$\begin{array}{c}\mathrm{p}=\frac{Number\;of\;literate\;girls\;in\;the\;age\;group\;13-19\;years\;in\;urban\;Varanasi}{Total\;girls\;in\;the\;age\;group\;13-19\;years\;in\;urban\;Varanasi}*100\\ \mathrm{p}=\frac{103373}{120986}*100\\ \mathrm{p}= 85.44\end{array}$$

The sample size estimation for the study is done by using the formula developed by Cochran.

(1977). The formula is as follows:

$$\mathrm{n}=\frac{\left(z\right)2*p*q}{\left(d\right)2}$$

where,

n = Required Sample Size; Z = 1.96 (95% level of confidence); p = 0.8544; q = 0.1456; and α = 0.05 (5% margin of error).

n = 191

By taking a non-response rate of 10 percent and a design effect of 1.5, the sample size was to be; n = 191*1.1*1.5 = 315 Individuals.

So, nearly 350 adolescent girls from the school were interviewed.

#### Sampling design

Varanasi district is subdivided into five zones for ease of administration namely; Aadampur Zone (20 wards), Bhelupur Zone (19 Wards), Kotwali Zone (13 Wards), Dashaswamedha Zone (21 Wards), and Varunapaar Zone (19 wards). Each zone is further divided into smaller segments known as wards. Cluster random sampling procedure was adopted to obtain the sample. Out of total five zones in Varanasi district, a total of ten schools were selected, two from each zone (Wards). Out of ten schools, five public and five private schools were selected. Two schools, one public and one private school was selected from each zone (wards). From each school, a total of 35 students were interviewed. These 35 students were selected from class 8^th^ to 12^th^. From each class, seven students were selected for the interview. The first author of this paper conducted all the interviews as this work is a part of her PhD project. The interviews were arranged at school and at household level. At first, a school was contacted and upon getting ethical clearance from school authorities and all the girls in selected classes were provided the informed consent form. All the girls were told about the purpose of the study and were asked to get the informed consent form signed from their mothers. Girls were also told that they shall inform that their mothers will also be interviewed at the respective house and should only sign the informed consent form if they intend to take part in this study. In short, the girl child was to be interviewed at school and mother at home. A detailed description of sampling procedure is provided below.

#### Selection of school

Varanasi city is divided into five zones, and zones are further divided into wards. One ward was selected from each zone randomly. After selecting five wards, one from each zone, a complete public, and private school listing was carried out. Two schools, one private and one public school, were randomly selected from each ward. If a ward does not have either of public or private school, the next ward was selected randomly. If in case a school is not interested in participating the study, the next school was selected randomly.

#### Selection of respondents from school

After receiving the informed consent form from the mothers, a list of all the eligible girls was prepared for respective class. From each class, seven girls were selected by employing systematic random sampling. After interviewing the girls, their mothers (adolescent girls) were personally interviewed at their respective households. It is to be noted that we proceeded for the informed consent form first and upon getting the informed consent form, we moved to select our sample using appropriate sampling procedure thereby negating chances of refusal or not getting response on interview.

#### Inclusion criteria

Girls aged 13–19 years of age and girls studying in class 8^th^ to 12^th^.were included in the study.

#### Exclusion criteria

Disabled girls and girls whose mothers were not alive were not part of the sampling procedure and such respondents were excluded while deriving the sampling frame.

#### Outcome variable

Self-rated health was the primary outcome variable of this study. SRH was categorized on a Likert scale ranging from 1 to 5, where 1,2, and 3 means Excellent, very good, and good, whereas, 4 and 5 means poor and very poor, respectively. For ease of analysis, SRH was categorized as a dichotomous variable where 0 means ‘Good SRH’ (comprising values 1,2, and 3) and 1 means ‘Poor SRH’ (comprising values 4 and 5). The exact wording of the question asked from the adolescent girls was, “In general, how would you say your health is?”.

#### Exposure variable

Exposure variables were divided into three groups namely; household characteristics, parental characteristics, and adolescent characteristics. Household Characteristics include; Caste [Scheduled Castes/Scheduled Tribes (SC/ST), Other Backward Class (OBC), and Others], Religion (Hindu and Non-Hindu), Wealth Index (Poorest, Poor, Middle, Rich, and Richest), and Composition of Children (Only daughter/no son, equal son and daughter, more son/less daughter, and more daughter/less son). Parental characteristics include; Father’s education level (No education, Primary, Secondary, Higher Secondary, and Higher Education), Mother’s education level (No education, up to primary, up to secondary, higher secondary, and graduation and above), Working status of father (Working and Not working), and Working status of mother (Working and Not working). Adolescent girl’s characteristics include; Girl’s education level (8^th^-10^th^ and 11^th^-12^th^) and Age of the girl (13–15 years and 16–19 years).

#### Creation of wealth index using principal component analysis (PCA)

There are several ways households' wealth or economic status, or living standards can be measured. A few of the most common of those measures include Income, Expenditure, and Consumption methods. The first two measures, i.e., Income and Expenditure, are hard to collect accurately. The best way is to use data on asset ownership and housing characteristics and combine this information into a proxy wealth indicator. Such indices are created through Principal Component Analysis (PCA) method. The benefit of using the asset ownership method is; it gives an indication of the longer-term economic status of a household and is less dependent on short-term economic changes. For this study, we have used asset ownership as a measure to create the wealth index.

The wealth index measures relative wealth and is not an absolute measure of poverty or wealth. For example, in an area where about 15% of all the households fall below the poverty line, 40% of the households will still fall into the two poorest quintiles and therefore be classified as poor when the whole population is divided into five quintiles.

For asset ownership measure, wealth is characterized by ownership of different types of assets in urban areas than in rural areas. Hence, wealth measures can be biased towards urban or rural households. The wealth Index created for this study is free from rural–urban biases as this study only has data from an urban set-up.

Supplementary file 1 presents the assets that were considered while creating the variable of wealth index. While starting with Principal Component Analysis (PCA), the 15 variables mentioned in supplementary file 1 were taken to create a wealth Index. After identifying these 15 variables, they require further investigation before opting for PCA. The rule of thumb is that if a variable (to be precise: Asset) is owned by more than 95% or less than 5% of the sample, it should be excluded from the analysis. The cot/bed and tractor availability were removed while creating a wealth index as around 97% of the households were having either a cot or bed and only 3 percent were having tractor. All variables were binary variables (where 1 indicates yes or availability and 0 indicates No or non-availability).

Once the variables were selected after preliminary check, PCA was run to create a wealth index. PCA is a data reduction technique that involves replacing many correlated variables with a set of principal uncorrelated ‘principal components’ that can explain much of the variance and represent the population's unobserved characteristics. The Kaiser–Meyer–Olkin Measure of sampling adequacy varies between 0 and 1. The values that are closer to 1 are better. A value of 0.6 is suggested as the minimum acceptable value. For this study, the KMO value was 0.879, which was entirely satisfactory to carry out the analysis. Bartlett’s test of sphericity was significant at 0.000 level with a chi-square value of 3456.21.

### Statistical analysis

The obtained data from the survey were processed (i.e., entry & editing) with the help STATA 13.1 package; later, cleaned data were analyzed using STATA -13.1 Package. The bivariate analysis was used to see the percentage/prevalence of SRH among respondents by various background characteristics. Bivariate analysis for categorical data was carried to understand the prevalence of poor SRH by various characteristics. Logistic regression analysis was carried out to report adjusted odds ratio by taking all the exposure variables in the model. The outcome variable was dichotomised to fit into the conditions of logistic regression. Our outcome variable was SRH which is dichotomous in nature. We have taken all the exposure variables in a single model to explore the odds ratio and did not take separate model for various types of exposure variables.

The results were presented in the form of odds ratio (OR) with a 95% confidence interval (CI).

The model is usually put into a more compact form as follows:

$$\mathrm{ln}\left(\frac{{P}_{i}}{1-{P}_{i}}\right)={\beta }_{0}+{\beta }_{1}{x}_{1}+\dots +{\beta }_{M}{x}_{m-1},$$

where ${\beta }_{0},\dots ..,{\beta }_{M}$ are regression coefficient indicating the relative effect of a particular explanatory variable on the outcome. These coefficients change as per the context in the analysis in the study.

### Ethical issues

The study proposal and survey questionnaires were approved by the Student Research Ethics Committee (SREC) of the institute. Written informed consent was taken from the individual respondents. Participation in the study was made voluntary, and participants were allowed to withdraw at any point during the interview if desired.

## Results

Table 1 depicts the background characteristic of adolescent girls. Almost one-fifth (19.7%) of the girls belonged to SC/ST caste, half (48.6%) of them belonged to OBC, and the remaining one-third (31.7%) belonged to Others caste group.

**Table 1:** Percentage distribution of the selected sample by various background characteristics

Background Characteristics	Total sample N (%)
**Household Characteristics**	
**Caste** SC/ST	69 (19.7)
OBC	170 (48.6)
Others	111 (31.7)
**Religion** Hindu	271 (77.4)
Non-Hindu	79 (22.6)
**Wealth Index**	
Poorest	67 (19.1)
Poor	73 (20.9)
Middle	70 (20.0)
Rich	69 (19.7)
Richest	71 (20.3)
**Composition of children**	
Only daughter/ no son	39 (11.1)
Equal son and daughter	126 (36.0)
More son/less daughter	117 (33.4)
More daughter/less son	68 (19.4)
**Parental Characteristics**	
**Father education**	
No education	53 (15.4)
Primary	54 (15.7)
Secondary	67 (19.4)
Higher Secondary	65 (18.8)
Higher education	106 (30.7)
**Mother education**	
No education	97 (27.7)
Primary	59 (16.9)
Secondary	78 (22.3)
Higher Secondary	83 (23.7)
Higher education	33 (9.4)
**Working status of Father**	
Working	334 (96.8)
Not working	11 (3.2)
**Working status of Mother**	
Working	39 (11.1)
Not working	311 (88.9)
**Adolescent girl’s characteristics**	
**Girl’s Education**	
8^th^-10^th^	210 (60.0)
11^th^-12^th^	140 (40.0)
**Age of the girl**	
13–15 years	181 (51.7)
16–19 years	169 (48.3)
**Total**	**350 (100)**

**Fig. 1:**
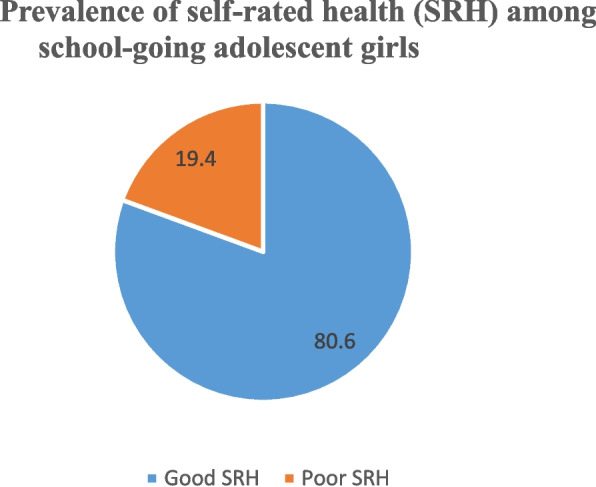
Prevalence of self rated health (SRH) among school going adolescent girls

Figure 1 shows the prevalence of self-rated health among adolescent girls. Almost one-fifth (19.4%) of the adolescent girls reported poor SRH. Supplementary file 2 provides information on self-rated health among adolescent girls where the outcome was measured on five-point Likert scale. Almost 42 percent of the girls reported their self-rated health as excellent and 13 percent reported their self-rated health as very poor. Table 2 depicts the prevalence of poor SRH among adolescent girls by various background characteristics. The prevalence of poor SRH was higher among adolescent girls belonging to SC/ST (34.8%), poorest households (35.8%), households with more number of daughters than number of sons (25%), and whose mothers had no education (27.8%). Table 3 shows the results of logistic regression analysis. Adolescent girls from OBC [OR: 0.39; 95% CI: 0.18-0.85] and Others caste [OR: 0.58; 95% CI: 0.23–0.87] were less likely to report poor SRH than their SC/ST counterparts. Household wealth index presented an interesting result discussed in detail in the discussion section. Girls who belonged to poor [OR: 0.62; 95% CI: 0.33–0.92] and middle [OR: 0.40; 95% CI: 0.13–0.72] household wealth quintile were less likely to report poor SRH than girls who belonged to the poorest households; however, insignificant results were noted among girls who belonged to rich and richest wealth category and in such scenario, odds could go either way. Nevertheless, this paradox has been discussed in detail in the discussion section of this paper. Composition of children in a household was a strong predictor of poor SRH among adolescent girls. Girls who belonged to a household with more number of daughters/less son were almost eight times [OR: 7.8; 95% CI: 1.5–39.5] more likely to report poor SRH than their counterparts.

**Table 2:** Prevalence of poor SRH as reported by school-going adolescent girls by various background characteristics

Background Characteristics	Poor SRH (%)
**Household Characteristics**	
**Caste**	
SC/ST	24 (34.8)
OBC	26 (15.3)
Others	18 (16.2)
**Religion**	
Hindu	52 (19.2)
Non-Hindu	16 (20.3)
**Wealth Index**	
Poorest	24 (35.8)
Poor	12 (16.4)
Middle	8 (11.4)
Rich	11 (15.9)
Richest	13 (18.3)
**Composition of children**	
Only daughter/ no son	2 (5.1)
Equal son and daughter	31 (24.6)
More son/less daughter	18 (15.4)
More daughter/less son	17 (25.0)
**Parental Characteristics**	
**Father education**	
No education	12 (22.6)
Primary	16 (29.6)
Secondary	11 (16.4)
Higher Secondary	10 (15.4)
Higher education	18 (17.0)
**Mother education**	
No education	27 (27.8)
Primary	6 (10.2)
Secondary	18 (23.1)
Higher Secondary	13 (15.7)
Higher education	4 (12.1)
Working status of Father*	
Working	63 (18.9)
Not working	4 (36.4)
**Working status of Mother**	
Working	8 (20.5)
Not working	60 (19.3)
**Adolescent girl’s characteristics**	
**Girl’s Education**	
8^th^-10^th^	41 (19.5)
11^th^-12^th^	27 (19.3)
**Age of the girl**	
13–15 years	36 (19.9)
16–19 years	32 (18.9)
**Total**	**68 (19.4)**

*means that the total may not add up to 68 as the case is with other categories, where total sums up to 68. Father of the one girl was not alive during the survey period and therefore, the sum adds up to 67 only

**Table 3:** Logistic regression analysis estimates for poor SRH among adolescent girls by various background characteristics

Background Characteristics	OR
**Household Characteristics**	
**Caste**	
SC/ST®	
OBC	0.39** (.18-.85)
Others	0.58** (.23–0.87)
**Religion**	
Hindu®	
Non-Hindu	0.95 (.47–1.9)
**Wealth Index**	
Poorest® Poor	0.62* (.33-0.92)
Middle	0.40* (.13–0.72)
Rich	0.44 (.15–1.3)
Richest	1.0 (.34–3.1)
**Composition of children**	
Only daughter/ no son®	
Equal son and daughter	8.4*** (1.8–39.9)
More son/less daughter	4.9** (1.1–24.5)
More daughter/less son	7.8*** (1.5–39.5)
**Parental Characteristics**	
**Father education**	
No education® Primary	2.4 (.82–6.8)
Secondary	1.00 (.31–3.2)
Higher Secondary	.99 (.30–3.3)
Higher education	1.50 (.43–5.3)
**Mother education**	
No education® Primary	.23** (.07-.75)
Secondary	.78 (.31–1.9)
Higher Secondary	.51 (.18–1.5)
Higher education	.21* (.04–1.1)
**Working status of Father**	
Working®	
Not working	1.9 (.45–7.7)
**Working status of Mother**	
Working®	
Not working	.48 (.16–1.5)
**Adolescent girl’s characteristics**	
**Girl’s Education**	
8th-10th®	
11th-12th	88* (.37–2.1)
**Age of the girl**	
13–15 years®	
16–19 years	84 (.36–1.9)

®: reference category; ***p < 0.01; **p < 0.05; *p < 0.1 *OR* Odds ratio, *CI* Confidence interval

## Discussion

This study explored the possible correlates of poor self-rated health among school-going adolescent girls. Literature that examined poor SRH among adults [27] and older adults [25, 26, 35] is abundantly present in the Indian context; however, minimal information is available examining the prevalence and correlates of poor SRH among adolescent girls in the urban setting of India. Almost one-fifth (19.4%) of the adolescent girls reported poor SRH. The prevalence of poor SRH in this study is higher from several previous studies conducted in different settings [2, 36]. The prevalence of poor SRH among adolescents aged 11–17 years in urban Brazil was almost 11 percent, whereas it was only 4 percent among the US adolescents [2]. The difference in the prevalence of poor SRH could be because of several reasons ranging from difference in the age group to gender and from the type of setting (urban–rural) to socioeconomic and demographic factors.

The girls from OBC and other caste groups were less likely to report poor SRH than girls from SC/ST caste group. The caste paradox is quite prevalent in Indian society. Scheduled Caste are generally considered backward and poor in terms of the resources they have. There is a significant disadvantage in education and economic status between SCs and others in the Indian social strata, especially for women [26]. Poor self-rated health among ST could be explained by social conditioning which creates the perception that SC/ST have lower expectations and are satisfied with their current health status [37, 38]. Moreover, other caste categories are better-off than SC/ST in terms of educational attainment, employment opportunities and so on. It is evident that SC/ST caste group has poor socioeconomic background [39]. Association between poor socioeconomic conditions and poor health is positively linked [39]; thereby, it can be inferred that a higher prevalence of poor SRH among SC/ST adolescent girls could be due to their poor socioeconomic backgrounds. Due to the deeply entrenched nature of social stratification of the caste system, which is ascribed at birth [40], it has created enormous social [41], educational [42], and economic [43] inequalities. Traditionally, lower castes (SC/ST) have been ostracized and stigmatized because they are located at the bottom of the hierarchy and historically have had less education, influence, and privileges than the upper castes. Additionally, women from marginalised groups are stigmatised, discriminated against and deprived of the best quality of life due to their position at the bottom of caste, class, and gender hierarchies [44]. As a result, ethnic discrimination permeates all aspects of life and contributes to poorer well-being outcomes [45].

In terms of socioeconomic background, the household's wealth quintile presented a finding worth discussing in the current context. Results revealed that adolescent girls from poor and middle wealth quintiles were less likely to report poor SRH than girls from poorest wealth quintile households; however, the results were insignificant for girls belonging to the rich and richest wealth quintile. How does wealth play its part in promoting SRH in this study is worth investing? Let us understand this paradox systematically. It is clear that adolescent girls from the poorest wealth quintile were more likely to report poor SRH. We have explained this; explained through a positive association between poverty and poor SRH [31]. What comes next is the relative household wealth that is critical in explaining poor SRH among adolescent girls. Girls from poor and middle wealth quintiles are poor but richer than girls from the poorest wealth quintile and therefore have fewer chances of reporting poor SRH. However, girls from the rich and richest wealth quintile could not replicate the mode of reporting SRH as their counterparts from the poor and middle wealth quintile. Possibly because SRH is also explained by the mother’s involvement in daughter’s life and girls from the poor and middle wealth quintile got emotional support from mothers as they were not working, whereas girls from rich and richest wealth quintile did not get that required support from mothers as they were working. Evidence across the countries has also suggested that mothers' involvement in their children's day-to-day life predicts SRH among their children [46].

Composition of children in a household was another predictor of poor SRH among adolescent girls. Girls were more likely to report poor SRH with any combination of number of sons and daughters in the household than when the household has no son. Previous studies have suggested that gender discrimination disfavouring girls is a bigger problem in health care utilization [47], which can rightly be attributed to their poor SRH [48]. Higher education among mothers acted as a safety net against poor SRH among girls, so was the education status of the girls. Previous studies have related higher educational status of the parents and adolescents to the good SRH [49]. In contrast, future educational aspirations have been linked to poor SRH among adolescents [50].

### Limitations of the study

The study findings shall be interpreted in light of the following limitations. The findings shall not be generalized in a greater context, to say at the state-level or national-level as the sample size is representative for Varanasi district only. Other limitation includes excluding girls without mothers and disabled girls.

## Conclusion

This study is important in the current context as minimal literature examining predictors of poor SRH among school-going adolescent girls in urban settings is available. The study confirmed that adolescent girls from SC/ST households were more likely to report poor SRH. Furthermore, composition of children was another important risk factor for girls reporting poor SRH. These findings call out for some policy suggestions. It is important to understand the involvement of mothers in improving SRH among adolescent girls as girls with any other composition of children in the family were more likely to report poor SRH than girls in the families with only daughters and no son.

### Supplementary Information

**Additional file 1: Supplementary file 1.** Variables included for creation of Wealth Index.

**Additional file 2: Supplementary file 2.** Prevalence of self-rated health among adolescent girls in Varanasi, India.

**Additional file 3: Supplementary file 3.** Interview Schedule for School-going Adolescent Girl.

**Additional file 4: Supplementary file 4.** Interview Schedule for Mothers.

## Data Availability

Data used are part of first author’s Ph.D. research work and can be made available upon reasonable request. The data request are to be made at- ratnapatelbhu@gmail.com.

## Abbreviations

OBC: Other Backward Classes
SC/ST: Scheduled Castes/Scheduled Tribes
SRH: Self-rated Health
