# Supplementary material for: Correlates of poor self-rated health among school-going adolescent girls in urban Varanasi, India
Source: BMC Public Health. 2023 Oct 4;23:1921. doi: 10.1186/s12889-023-16822-1 (PMC10552224; doi:10.1186/s12889-023-16822-1)
Supplement: Supplementary file 1 — Additional file 1: Supplementary file 1. Variables included for creation of Wealth Index. [file 12889_2023_16822_MOESM1_ESM.pdf]

Supplementary file 1: Variables included for creation of Wealth Index

S. No.	Assets	% (N)
1	Cot/Bed	97.14 (340)
2	Table	82.86 (290)
3	Electric fan	84.29 (295)
4	Radio	23.14 (81)
5	TV (Either B&W or colour TV)	92.00 (322)
6	Sewing machine	36.29 (127)
7	Telephone/Mobile	94.28 (330)
8	Computer/laptop	21.14 (74)
9	Refrigerator	50.00 (175)
10	Cooler/AC	47.71 (167)
11	Washing machine	38.29 (134)
12	Bicycle	76.86 (269)
13	Motorcycle	82.00 (287)
14	Car	14.57 (51)
15	Tractor	3.14 (11)
