# Supplementary material for: Correlates of poor self-rated health among school-going adolescent girls in urban Varanasi, India
Source: BMC Public Health. 2023 Oct 4;23:1921. doi: 10.1186/s12889-023-16822-1 (PMC10552224; doi:10.1186/s12889-023-16822-1)
Supplement: Supplementary file 2 — Additional file 2: Supplementary file 2. Prevalence of self-rated health among adolescent girls in Varanasi, India. [file 12889_2023_16822_MOESM2_ESM.pdf]

Supplementary file 2: Prevalence of self-rated health among adolescent girls in Varanasi, India		
Self-rated health	Percentage	N
Excellent	41.7	146
Very Good	18.6	65
Good	20.3	71
Poor	6.3	22
Very Poor	13.1	46
Total	100	350
