# Supplementary material for: Correlates of poor self-rated health among school-going adolescent girls in urban Varanasi, India
Source: BMC Public Health. 2023 Oct 4;23:1921. doi: 10.1186/s12889-023-16822-1 (PMC10552224; doi:10.1186/s12889-023-16822-1)
Supplement: Supplementary file 3 — Additional file 3: Supplementary file 3. Interview Schedule for School-going Adolescent Girl. [file 12889_2023_16822_MOESM3_ESM.pdf]

__Supplementary file 3: Interview Schedule for School-going Adolescent Girl__

SECTION I: PERSONAL INFORMATION:

S. No.	Questions	Coding Categories	Skip/<br>Go to
1.	What is your date of birth?	1.A Day of Birth..........|___|___| 1.B Month of birth .....|___|___| 1.C Year of Birth ....|___|___|___|___| Don't Know...................................... 98	
2.	How old were you on your last birthday?<br>Compare and correct 1 and 2, if inconsistent	Age in completed years ....|___|___|	
3.	In which class are you studying?	8th... .. 1<br>9th.. 2<br>10th .... 3<br>11th .... 4<br>12th 5	
4.	Years of schooling	7 to 15 years ....|___|___	
5.	How many brothers and sisters you have?	Brothers ....|___|___| Sisters ....|___|___| No sibling.................................... 98	→ Skip to 9
6.	Are you the eldest of all of your siblings?	Yes... 1<br>No... 2	
7.	How many of your siblings are elder to you?<br>**(Do not answer if you are the eldest)**	Brother …………………..<br>Sister …………………….	
8.	Educational Status of each of your siblings	S. No. Brother/Sister Class<br>1<br>2<br>3<br>4<br>5<br>6	
**Mass Media Exposure**			
9.	Do you read a newspaper or magazine?	Almost every day .... 1<br>At least once a week .... 2<br>Not at all 3	
10.	Do you listen to radio?	Almost every day .... 1<br>At least once a week .... 2<br>Not at all 3	
11.	Do you watch television?	Almost every day .... 1<br>At least once a week .... 2<br>Not at all .. 3	
12.	Do you go to movies/theatre?	At least once a week .... 1<br>At least once a month .... 2<br>Not at all 3	
13.	Do you own a mobile?	Yes .... 1<br>No 2	
14.	Are you active on social media platforms?	Yes .... 1<br>No 2	
15.	How much time do you spend on social media per day?	Less than one hour .... 1<br>More than one hour 2	
16.	Name of the school	....<br>..	

17.	Type of school	Government .... 1<br>Private .... 2<br>Trust .... 3<br>Municipal corporation .... 4<br>Religious 5	
18.	Nature of the school	Girls only .... 1<br>Co-education 2	
19.	Medium taught in the school	Hindi .... 1<br>English .... 2<br>Other Language 3	
20.	Syllabus followed	State .... 1<br>CBSE .... 2<br>ICSE .... 3<br>Any Other.................................... 98	
21.	Religion	Hindu .... 1<br>Muslim .... 2<br>Christian .... 3<br>Other religion 4	
22.	Mother's education	Illiterate .... 1<br>Literate but no education .... 2<br>Primary .... 3<br>Secondary..................................... 4<br>Higher 5	
23.	Father's education	Illiterate .... 1<br>Literate but no education .... 2<br>Primary .... 3<br>Secondary .... 4<br>Higher 5	
24.	Occupation of Mother	Government job........................... 1<br>Private job .... 2<br>Self-employed .... 3<br>House-wife .... 4<br>Other (Specify)...........................<br>.. 98	
25.	Occupation of father	Government job .... 1<br>Private job .... 2<br>Self-employed .... 3<br>No job .... 4<br>Other (Specify)...........................<br>.. 98	
26.	Information regarding working parents	One working at distance place ..... 1<br>Both working at distance place .... 2<br>One working at nearby distance ... 3<br>Both working at nearby 4	
27	Which type of family are you coming from?	Nuclear Family .... 1<br>Joint Family .... 2<br>Extended family........................ 3	

SECTION II: SOCIAL CAPITAL VARIABLES

S. No.	Questions	Coding Categories	Skip/ Go to
**FAMILY SOCIAL CAPITAL**			
28	Do you feel that your family understand and give attention to you?	Strongly Agree .... 1<br>Agree .... 2<br>Neither Agree nor Disagree .... 3<br>Disagree .... 4<br>Strongly Disagree 5	

29	give attention to you?<br>generally receive you at home?	Any of the parents .... 1<br>Grandparents .... 2<br>Any of the realtive .... 3<br>No one 4	
30	Everyone in my family loves me and understands me	Strongly Agree .... 1<br>Agree .... 2<br>Neither Agree nor Disagree ..... 3<br>Disagree .... 4<br>Strongly Disagree.................... 5	
31	In my family, we talk about important things	Strongly Agree .... 1<br>Agree .... 2<br>Neither Agree nor Disagree ..... 3<br>Disagree .... 4<br>Strongly Disagree.................... 5	
32	In my family, we listen to each other	Strongly Agree .... 1<br>Agree .... 2<br>Neither Agree nor Disagree ..... 3<br>Disagree .... 4<br>Strongly Disagree.................... 5	
33	In my family, we support each other	Strongly Agree .... 1<br>Agree .... 2<br>Neither Agree nor Disagree ..... 3<br>Disagree .... 4<br>Strongly Disagree.................... 5	
34	In my family, we resolve misunderstanding	Strongly Agree .... 1<br>Agree .... 2<br>Neither Agree nor Disagree ..... 3<br>Disagree .... 4<br>Strongly Disagree.................... 5	
35	In my family, we have dinner together	Strongly Agree .... 1<br>Agree .... 2<br>Neither Agree nor Disagree ..... 3<br>Disagree .... 4<br>Strongly Disagree.................... 5	
36	In my family, we generally go out for movies/picnic/dinner together on a regular basis	Strongly Agree .... 1<br>Agree .... 2<br>Neither Agree nor Disagree ..... 3<br>Disagree .... 4<br>Strongly Disagree.................... 5	
**NEIGHBOURHOOD SOCIAL CAPITAL**			
37	Do you feel people trust each other in your neighbourhood?	Strongly Agree .... 1<br>Agree .... 2<br>Neither Agree nor Disagree ..... 3<br>Disagree .... 4<br>Strongly Disagree.................... 5	
38	Do you feel that your neighbours step in to criticise someone's deviant behaviour during school?	Strongly Agree .... 1<br>Agree .... 2<br>Neither Agree nor Disagree ..... 3<br>Disagree .... 4<br>Strongly Disagree.................... 5	
39	In my neighbourhood, I feel safe	Strongly Agree .... 1<br>Agree .... 2<br>Neither Agree nor Disagree ..... 3<br>Disagree .... 4<br>Strongly Disagree.................... 5	
**SCHOOL SOCIAL CAPITAL**			
40	Do you feel teachers and students trust each other in your school? (Vertical School Trust)	Strongly Agree .... 1<br>Agree 2	

		Neither Agree nor Disagree ..... 3<br>Disagree .... 4<br>Strongly Disagree.................... 5	
41	Do you feel students trust each other in your school? (Horizontal School Trust)	Strongly Agree .... 1<br>Agree .... 2<br>Neither Agree nor Disagree ..... 3<br>Disagree .... 4<br>Strongly Disagree.................... 5	
42	Do you feel student collaborate with each other in your school? (Reciprocity at school)	Strongly Agree .... 1<br>Agree .... 2<br>Neither Agree nor Disagree ..... 3<br>Disagree .... 4<br>Strongly Disagree.................... 5	
43	I feel that my teachers appreciate me	Strongly Agree .... 1<br>Agree .... 2<br>Neither Agree nor Disagree ..... 3<br>Disagree .... 4<br>Strongly Disagree.................... 5	
44	I feel that our teachers treat students fairly	Strongly Agree .... 1<br>Agree .... 2<br>Neither Agree nor Disagree ..... 3<br>Disagree .... 4<br>Strongly Disagree.................... 5	
45	I feel that my teacher understand my problems	Strongly Agree .... 1<br>Agree .... 2<br>Neither Agree nor Disagree ..... 3<br>Disagree .... 4<br>Strongly Disagree.................... 5	
46	I feel our teachers acknowledge and respect student's own opinion	Strongly Agree .... 1<br>Agree .... 2<br>Neither Agree nor Disagree ..... 3<br>Disagree .... 4<br>Strongly Disagree.................... 5	
47	I usually get along well with my teachers	Strongly Agree .... 1<br>Agree .... 2<br>Neither Agree nor Disagree ..... 3<br>Disagree .... 4<br>Strongly Disagree.................... 5	
48	How do you feel about school at present	Excellent .... 1<br>Good.. 2<br>Fair... 3<br>Poor.. 4<br>Very poor................................ 5<br>Other (specify) 98	
**Relationship with classmates using three items**			
49	Students enjoy being together	Strongly Agree .... 1<br>Agree .... 2<br>Neither Agree nor Disagree ..... 3<br>Disagree .... 4<br>Strongly Disagree.................... 5	
50	Most students are kind and helpful	Strongly Agree .... 1<br>Agree .... 2<br>Neither Agree nor Disagree ..... 3<br>Disagree .... 4<br>Strongly Disagree.................... 5	
51	Students accept me as I am	Strongly Agree .... 1<br>Agree .... 2<br>Neither Agree nor Disagree ..... 3<br>Disagree 4	

		Strongly Disagree....................	5	
**Students- Teachers Relationship using three items scale**				
52	Teachers accept me as I am	Strongly Agree ....<br>Agree ....<br>Neither Agree nor Disagree .....<br>Disagree ....<br>Strongly Disagree....................	1<br>2<br>3<br>4<br>5	
53	Teachers care about me	Strongly Agree ....<br>Agree ....<br>Neither Agree nor Disagree .....<br>Disagree ....<br>Strongly Disagree....................	1<br>2<br>3<br>4<br>5	
54	I feel a lot of trust in my teachers	Strongly Agree ....<br>Agree ....<br>Neither Agree nor Disagree .....<br>Disagree ....<br>Strongly Disagree....................	1<br>2<br>3<br>4<br>5	
55	State the facilities in your school	Adequate teaching Staff ....<br>Proper Class room ....<br>Availability of basic amenities...<br>(drinking water, washrooms)<br>Playground.................................<br>Playing equipments ....<br>Library ....<br>Lab Facilities ....<br>Good school environment Extra<br>Curricular Activities	1<br>2<br>3<br><br>4<br>5<br>6<br>7<br>8<br>9	
56	Do you satisfied with the facilities available at your school?	Yes ....<br>No	1<br>2	

SECTION *III*: HEALTH, WELL-BEING AND SOCIAL CAPITAL

S. No.	Questions	Coding Categories		Skip/ Go to
57	Do you think that your parents feel worried when you get ill?	Yes ....<br>No	1<br>2	
58	Do you think that your other siblings get better treatment when they fell ill?	Yes ....<br>No	1<br>2	
59	Do you have any hospital in your nearby area?	Yes ....<br>No	1<br>2 →	Skip to 61
60	If yes, type of hospital	Private Hospital ....<br>Public Hospital ....<br>Medical Collage.................<br>Clinics...............................<br>Charity run Hospital	1<br>2<br>3<br>4<br>5	
61	When you fell ill, your parents prefer to take you to which type of hospital?	Private Hospital ....<br>Public Hospital ....<br>Medical Collage.................<br>Clinics...............................<br>Charity run Hospital ....<br>Medicine from shop...........<br>Other (specify)__________<br>Did Nothing......................	1<br>2<br>3<br>4<br>5<br>6<br>96<br>98	
62	The hospital you visit, when you fell ill, is generally how far from your home?	Within 5 km ....<br>Within 10 km ....<br>Within 15 km ....<br>Within 20 km ....<br>Within 25 km ....<br>More than 25 km	1<br>2<br>3<br>4<br>5<br>6	

		Don't know	98	
63	The hospital your other siblings visit, when they fell ill, is generally how far from your home?	Within 5 km ....<br>Within 10 km ....<br>Within 15 km ....<br>Within 20 km ....<br>Within 25 km ....<br>More than 25 km ....<br>Don't know.......................	1<br>2<br>3<br>4<br>5<br>6<br>98	
64	Do you think that in your neighbourhood there is no any good hospital?	Yes ....<br>No	1<br>2	
65	Do you know any good hospital which is not very near to your home?	Yes ....<br>No	1<br>2	
66	Did you ever fell seriously ill?	Yes ....<br>No	1<br>2	
67	Have you ever visited that good hospital when you fell seriously ill?	Yes ....<br>No	1<br>2	
68	Do your other siblings visited that good hospital when they fell seriously ill?	Yes ....<br>No	1<br>2	
69	Do the boys in your home visit better hospital (in terms of facilities) than girls at your home?	Yes ....<br>No	1<br>2	
70	Your neighbours show concern when you fell ill?	Yes ....<br>No	1<br>2	
71	Do the people from your neighbourhood come to meet you when you fell ill?	Yes ....<br>No	1<br>2	
72	Will your neighbours lend your parents some money, if they need to cover medical expenses?	Yes ....<br>No	1<br>2	
73	Do you think that your school has got the basic medical facilities covered?	Yes ....<br>No	1<br>2	
74	Do you feel that your school authorities show prompt response when you do not feel good in terms of your health?	Yes ....<br>No	1<br>2	
75	Does school authority understood your health concern and act accordingly?	Yes ....<br>No	1<br>2	

SECTION IV: EDUCATIONAL OUTCOMES, EDUCATIONAL ASPIRATIONS, FUTURE GOALS, AND SOCIAL CAPITAL

S. No.	Questions	Coding Categories		Skip/ Go to
76	Do you want to pursue higher study after school?	Yes ....<br>No	1<br>2	If no, skip the section
77	Will your parents allow you to pursue higher study?	Yes ....<br>No	1<br>2	
78	Are your parents supportive for your study?	Yes ....<br>No	1<br>2	
79	Does your parents have high hopes on you for your educational achievements?	Yes ....<br>No	1<br>2	
80	Do you feel pressure of achievement in study because of your parents?	Yes ....<br>No	1<br>2	
81	Does your school teachers motivate you for you good performance in school?	Yes ....<br>No	1<br>2	
82	Do you have a future goal in your life?	Yes ....<br>No	1<br>2 →	If no, skip to 86
83	Have you ever discussed your future goal with your parents?	Yes ....<br>No	1<br>2	
84	Does your parents are supportive for your future goal?	Yes ....<br>No	1 →<br>2	If yes, skip 86
85	Why parents are not supportive for your future	....		

	goal? Expain	....	
86	Does your teachers understand your educational capacity and act accordingly to that?	Yes .... 1<br>No 2	

SECTION V: GIRL'S AUTONOMY AND SOCIAL CAPITAL

S. No.	Questions	Coding Categories	Skip/ Go to
87	Do you say that you enjoy personal space at home?	Yes .... 1<br>No 2	
88	Are you free to choose which stream you will choose after your intermediate?	Yes .... 1<br>No 2	
89	Do you feel that your brothers are enjoying more freedom than you?	Yes .... 1<br>No 2	
90	Your parents try to impose their wishes on you for your academic decisions?	Yes .... 1<br>No 2	
91	Do you go for tuition?	Yes .... 1<br>No .... 2 →	Skip to 93
92	If yes, who decides the tutor?	Self .... 1<br>Parents .... 2<br>You along with parents .... 3<br>Your neighbours .... 4<br>Others (specify)_________ 98	
93	Does tutor comes to your home or you go to his/her place for tuition?	Tutor comes to home .... 1<br>I go to tutor's home .... 2<br>I go to tutor's coaching..... 3<br>centre	
94	Are you free to decide your Academic decisions?	Yes .... 1<br>No 2	
95	Do anyone stop you for roaming around your neighbourhood as per your wishes?	Yes .... 1<br>No 2	

SECTION VI: KIDCREEN 52 QUESTIONNAIRE

**SECTION 1: Physical activities and Health**		
96	In General, How would you say your health is?	Excellent .... 1<br>Very Good .... 2<br>Good .... 3<br>Poor .... 4<br>Very Poor 5
97	Thinking about last week:<br>Have you felt physically fit and well?	Not at all...................... 1<br>Slightly......................... 2<br>Moderately................... 3<br>Very............................. 4<br>Extremely.................... 5
98	Thinking about last week:<br>Have you been physically active (e.g. running, climbing, cycling)?	Not at all...................... 1<br>Slightly......................... 2<br>Moderately................... 3<br>Very............................. 4<br>Extremely.................... 5
99	Thinking about last week:<br>Have you been able to run well?	Not at all...................... 1<br>Slightly......................... 2<br>Moderately................... 3

		Very.............................<br>Extremely...................	4<br>5
100	Thinking about last week:<br>Have you felt full of energy?	Never...........................<br>Almost never..............<br>Sometimes.................<br>Almost always............<br>Always.......................	1<br>2<br>3<br>4<br>5
**Section 2: Feelings**			
101	Thinking about last week:<br>Have your life been enjoyable?	Not at all.....................<br>Slightly.........................<br>Moderately..................<br>Very.............................<br>Extremely...................	1<br>2<br>3<br>4<br>5
102	Thinking about last week:<br>Have you felt pleased that you are alive?	Not at all.....................<br>Slightly.........................<br>Moderately..................<br>Very.............................<br>Extremely...................	1<br>2<br>3<br>4<br>5
103	Thinking about last week:<br>Have you felt satisfies with your life?	Not at all.....................<br>Slightly.........................<br>Moderately..................<br>Very.............................<br>Extremely...................	1<br>2<br>3<br>4<br>5
104	Thinking about last week:<br>Have you been in good mood?	Never...........................<br>Almost never..............<br>Sometimes.................<br>Almost always............<br>Always.......................	1<br>2<br>3<br>4<br>5
105	Thinking about last week:<br>Have you felt Cheerful?	Never...........................<br>Almost never..............<br>Sometimes.................<br>Almost always............<br>Always.......................	1<br>2<br>3<br>4<br>5
106	Thinking about last week:<br>Have you had fun?	Never...........................<br>Almost never..............<br>Sometimes.................<br>Almost always............<br>Always.......................	1<br>2<br>3<br>4<br>5
**Section 3: General Mood**			
107	Thinking about last week:<br>Have you felt that you do everything badly?	Never...........................<br>Almost never..............<br>Sometimes.................<br>Almost always............<br>Always.......................	1<br>2<br>3<br>4<br>5
108	Thinking about last week:<br>Have you felt sad?	Never...........................<br>Almost never..............<br>Sometimes.................<br>Almost always............<br>Always.......................	1<br>2<br>3<br>4<br>5
109	Thinking about last week:<br>Have you felt so bad that you didn’t want to do anything?	Never...........................<br>Almost never..............<br>Sometimes.................<br>Almost always............	1<br>2<br>3<br>4

		Always.......................	5
110	Thinking about last week:<br>Have you felt that everything in your life goes wrong?	Never..........................<br>Almost never..............<br>Sometimes.................<br>Almost always............<br>Always.......................	1<br>2<br>3<br>4<br>5
111	Thinking about last week:<br>Have you felt fed up?	Never..........................<br>Almost never..............<br>Sometimes.................<br>Almost always............<br>Always.......................	1<br>2<br>3<br>4<br>5
112	Thinking about last week:<br>Have you felt lonely?	Never..........................<br>Almost never..............<br>Sometimes.................<br>Almost always............<br>Always.......................	1<br>2<br>3<br>4<br>5
113	Thinking about last week:<br>Have you felt under pressure?	Never..........................<br>Almost never..............<br>Sometimes.................<br>Almost always............<br>Always.......................	1<br>2<br>3<br>4<br>5
**Section 4: About Yourself**			
114	Thinking about last week:<br>Have you been happy with the way you are?	Never..........................<br>Almost never..............<br>Sometimes.................<br>Almost always............<br>Always.......................	1<br>2<br>3<br>4<br>5
115	Thinking about last week:<br>Have you been happy with your clothes?	Never..........................<br>Almost never..............<br>Sometimes.................<br>Almost always............<br>Always.......................	1<br>2<br>3<br>4<br>5
116	Thinking about last week:<br>Have you been worried about the way you look?	Never..........................<br>Almost never..............<br>Sometimes.................<br>Almost always............<br>Always.......................	1<br>2<br>3<br>4<br>5
117	Thinking about last week:<br>Have you felt jealous of the way other girls look?	Never..........................<br>Almost never..............<br>Sometimes.................<br>Almost always............<br>Always.......................	1<br>2<br>3<br>4<br>5
118	Thinking about last week:<br>Would you like to change something about your body?	Never..........................<br>Almost never..............<br>Sometimes.................<br>Almost always............<br>Always.......................	1<br>2<br>3<br>4<br>5
**Section 5: Free Time**			
119	Thinking about last week:<br>Have you had enough time for yourself?	Never..........................<br>Almost never..............<br>Sometimes.................<br>Almost always............<br>Always.......................	1<br>2<br>3<br>4<br>5

120	Thinking about last week:<br>Have you been able to do the things that you want to do in your free time?	Never.......................... 1<br>Almost never.............. 2<br>Sometimes................. 3<br>Almost always............ 4<br>Always....................... 5
121	Thinking about last week:<br>Have you had enough opportunity to be outside?	Never.......................... 1<br>Almost never.............. 2<br>Sometimes................. 3<br>Almost always............ 4<br>Always....................... 5
122	Thinking about last week:<br>Have you had enough time to meet friends?	Never.......................... 1<br>Almost never.............. 2<br>Sometimes................. 3<br>Almost always............ 4<br>Always....................... 5
123	Thinking about last week:<br>Have you been able to choose what to do in your free time?	Never.......................... 1<br>Almost never.............. 2<br>Sometimes................. 3<br>Almost always............ 4<br>Always....................... 5
**Section 6: Family and Home Life**		
124	Thinking about last week:<br>Have your parents understood you?	Not at all..................... 1<br>Slightly........................ 2<br>Moderately.................. 3<br>Very............................. 4<br>Extremely................... 5
125	Thinking about last week:<br>Have you felt loved by your parents?	Not at all..................... 1<br>Slightly........................ 2<br>Moderately.................. 3<br>Very............................. 4<br>Extremely................... 5
126	Thinking about last week:<br>Have you been happy at home?	Never.......................... 1<br>Almost never.............. 2<br>Sometimes................. 3<br>Almost always............ 4<br>Always....................... 5
127	Thinking about last week:<br>Have your parents had enough time for you?	Never.......................... 1<br>Almost never.............. 2<br>Sometimes................. 3<br>Almost always............ 4<br>Always....................... 5
128	Thinking about last week:<br>Have your parents treated you fairly?	Never.......................... 1<br>Almost never.............. 2<br>Sometimes................. 3<br>Almost always............ 4<br>Always....................... 5
129	Thinking about last week:<br>Have you been able to talk to your parents when you wanted to?	Never.......................... 1<br>Almost never.............. 2<br>Sometimes................. 3<br>Almost always............ 4<br>Always....................... 5
**Section 7: Money Matters**		
130	Thinking about last week:	Never.......................... 1

	Have you had enough money to do the same things as your friends do?	Almost never.............. 2<br>Sometimes................. 3<br>Almost always............ 4<br>Always....................... 5
131	Thinking about last week:<br>Have you had enough money for your expenses?	Never.......................... 1<br>Almost never.............. 2<br>Sometimes................. 3<br>Almost always............ 4<br>Always....................... 5
132	Thinking about last week:<br>Do you have enough money to do things with your friends?	Not at all..................... 1<br>Slightly......................... 2<br>Moderately.................. 3<br>Very............................. 4<br>Extremely................... 5
**Section 8: Friends**		
133	Thinking about last week:<br>Have you spent time with your friends?	Never.......................... 1<br>Almost never.............. 2<br>Sometimes................. 3<br>Almost always............ 4<br>Always....................... 5
134	Thinking about last week:<br>Have you done things with other girls?	Never.......................... 1<br>Almost never.............. 2<br>Sometimes................. 3<br>Almost always............ 4<br>Always....................... 5
135	Thinking about last week:<br>Have you had fun with your friends?	Never.......................... 1<br>Almost never.............. 2<br>Sometimes................. 3<br>Almost always............ 4<br>Always....................... 5
136	Thinking about last week:<br>Have you and your friends helped each other?	Never.......................... 1<br>Almost never.............. 2<br>Sometimes................. 3<br>Almost always............ 4<br>Always....................... 5
137	Thinking about last week:<br>Have you been able to talk about everything with your friends?	Never.......................... 1<br>Almost never.............. 2<br>Sometimes................. 3<br>Almost always............ 4<br>Always....................... 5
138	Thinking about last week:<br>Have you been able to rely on your friends?	Never.......................... 1<br>Almost never.............. 2<br>Sometimes................. 3<br>Almost always............ 4<br>Always....................... 5
**Section 9: School and Learning**		
139	Thinking about last week:<br>Have you been happy at school?	Not at all..................... 1<br>Slightly......................... 2<br>Moderately.................. 3<br>Very............................. 4<br>Extremely................... 5
140	Thinking about last week:	Not at all..................... 1

	Have you got on well at school?	Slightly......................... 2<br>Moderately.................. 3<br>Very.............................. 4<br>Extremely................... 5
141	Thinking about last week:<br>Have you been satisfied with your teachers?	Not at all..................... 1<br>Slightly......................... 2<br>Moderately.................. 3<br>Very.............................. 4<br>Extremely................... 5
142	Thinking about last week:<br>Have you been able to pay attention?	Never.......................... 1<br>Almost never.............. 2<br>Sometimes................. 3<br>Almost always............ 4<br>Always....................... 5
143	Thinking about last week:<br>Have you enjoyed going to school?	Never.......................... 1<br>Almost never.............. 2<br>Sometimes................. 3<br>Almost always............ 4<br>Always....................... 5
144	Thinking about last week:<br>Have you got along well with your teachers?	Never.......................... 1<br>Almost never.............. 2<br>Sometimes................. 3<br>Almost always............ 4<br>Always....................... 5
**Section 10: Bullying**		
145	Thinking about last week:<br>Have you been afraid of other girls and boys?	Never.......................... 1<br>Almost never.............. 2<br>Sometimes................. 3<br>Almost always............ 4<br>Always....................... 5
146	Thinking about last week:<br>Have other girls and boys made fun of you?	Never.......................... 1<br>Almost never.............. 2<br>Sometimes................. 3<br>Almost always............ 4<br>Always....................... 5
147	Thinking about last week:<br>Have other girls and boys bullied you?	Never.......................... 1<br>Almost never.............. 2<br>Sometimes................. 3<br>Almost always............ 4<br>Always....................... 5
