# Supplementary material for: Correlates of poor self-rated health among school-going adolescent girls in urban Varanasi, India
Source: BMC Public Health. 2023 Oct 4;23:1921. doi: 10.1186/s12889-023-16822-1 (PMC10552224; doi:10.1186/s12889-023-16822-1)
Supplement: Supplementary file 4 — Additional file 4: Supplementary file 4. Interview Schedule for Mothers. [file 12889_2023_16822_MOESM4_ESM.pdf]

Supplementary file 4: Interview Schedule for Mothers

Section I: Household Information

I would like to have some information about the people who usually live in your household

LINE NO 001	USUAL RESIDENTS OF THE HH	RELATION SHIP WITH THE HEAD OF HH	SEX	AGE	MARITAL STATUS	EDUCATION (if age >5 years)	WORKING STATUS		
	Please tell me the names of the persons who usually live in your HH starting with the head of the HH	What is the relationship of (Name) to the head of the household?	Is (Name) male or female? 01= Male 02= Female 03= Others	How old is (Name)? (In Completed Years)	What is the current marital status of (Name)?	What is the highest standard (Name) has completed?	What is the current working status of (Name)? 1= Working 2= Not Working	Is (Name) in full time or part time employment (If working) 1 = Yes 2 = No	What is the nature of employment (if working)
(1)	(2)	(3)	(4)	(5)	(6)	(7)	(8)	(9)	(10)
1.									
2.									
3.									
4.									
5.									
6.									
7.									
8.									
9.									
10.									
11.									
12.									

Please add sheets and columns if more members are in the household.

Codes for Q. 3	Codes for Q. 6	Codes for Q. 7	Codes for Q. 10
01= Head	01= Currently Married	01= No education<br>02= No education, but can read and write	01 = Government Sector
02= Husband or wife	02= Widowed	03= Primary	02 = Private Sector
03= Son or Daughter	03= Divorced	04= Secondary	03 = Self-employed/ Business
04= Son-in-law or Daughter-in-law	04= Separated	05= Higher Secondary	04 = Daily wage worker
05= Grand Child	05= Never Married	06= Graduation	05 = Agriculture
06= Brother or Sister		07= Post Graduation	06 = Household work
07= Niece/ Nephew		08= Professional	98 = other (Specify)
08= Other Relatives			

S. NO.	Questions	Coding categories	Skip/ Go to
18.	What is the religion of the head of the household	Hindu .... 1<br>Muslim.. 2<br>Other (Specify) .... 96<br>Don't Know 98	
19.	What is the Caste of the head of the household	Scheduled caste .... 1<br>Scheduled Tribe .... 2<br>Other Backward .... 3<br>Other (Specify) .... 4<br>Don't Know 98	
20.	What is the language you generally speak at home	Hindi .... 1<br>English .... 2<br>Urdu... 3<br>Bangali.. 4<br>Punjabi... 5<br>Other (Specify) 98	
21.	Have you always lived in this city?	Yes .... 1<br>No 2	
22.	How long have you been living in this city?	Months |___|___| Years |___|___|___|___| Don't Know 98	
23.	Note the type of house (Observe and Record)	Kuccha House .... 1<br>Semi Pucca house.................................. 2<br>Pucca House 3	
24.	How many rooms are there in your home?<br>(excluding bathrooms, balconies, or hallways but including kitchen)	Number of rooms .... [ ]	
25.	Of these rooms, how many are used for sleeping?	Number of rooms .... [ ]	
26.	Do you have separate room for kitchen?	Yes .... 1<br>No 2	
27.	What is the main source of drinking water for the household?	Piped water .... 1<br>Water from spring .... 2<br>Rain water .... 3<br>Tanker .... 4<br>Bottled water/ purchased water .... 5<br>Hand pump .... 6<br>Tube well/ bore well .... 7<br>Other (Specify) 98	
28.	Does your household have	Yes No<br>Electricity .... 1 2<br>A cot or bed .... 1 2<br>A table .... 1 2<br>An electric fan .... 1 2<br>Radio .... 1 2<br>Black & white TV .... 1 2<br>Colour TV... 1 2<br>Sewing Machine .... 1 2<br>Telephone .... 1 2<br>Mobile 1 2	

		Computer/ Laptop .... 1 2<br>Refrigerator .... 1 2<br>AC/ Cooler .... 1 2<br>Washing Machine .... 1 2<br>Bicycle .... 1 2<br>Motor Cycle .... 1 2<br>Car .... 1 2<br>Tractor 1 2	
29.	What type of fuel does HH mainly use for cooking	Electricity .... 1<br>LPG/ Natural Gas .... 2<br>Biogas .... 3<br>Kerosene .... 4<br>Coal .... 5<br>Wood .... 6<br>Agriculture Waste .... 7<br>Dung cakes .... 8<br>Others (Specify)______________________ 98	
30.	What type of toilet facility do members of your household use?	Flush Toilet .... 1<br>Pit latrine with slab .... 2<br>Pit latrine without slab .... 3<br>Use open space .... 4<br>Other (Specify) ____________________ 98	
31.	Does any member of this household own this house	Yes... 1<br>No.. 2 →	skip to 33
32.	Who owns this house (Write S. No. from Q. 1 in household roaster)	S. No ( )	
33.	Does your household have any agricultural land?	Yes.. 1<br>No... 2 →	Skip to36
34.	How much? (acres/ beegha, mention)	---	
35.	Who owns this land (Write S. No. from Q. 101 in household roaster)	S. No ( )	
36.	What is the main source of lighting in your house?	Electricity 1<br>Kerosene 2<br>Gas 3<br>Solar energy 4<br>Others (specify) 98	
37.	Does your household has ration card?	Yes 1<br>No 2 →	Skip to 39
38.	Colour of ration card	Yes No<br>Yellow (BPL) 1 2<br>Yellow (Antyodaya scheme) 1 2<br>Saffron (APL; PatrGrahasthi) 1 2<br>Others Specify 98 98	
39.	What is the total monthly income of your household? (In Rs.)	Below 5000 1<br>5001 – 10000 2<br>10001 – 20000 3<br>20001 – 50000 4<br>50001- 100000 5<br>Above 1 Lakh 6	

SECTION II: PERSONAL INFORMATION:

S. No.	Questions	Coding Categories		Skip/ Go to
40.	How old were you on your last birthday?	Age in completed years|___|___|		
41.	What is your current marital status?	Married<br>Separated<br>Widowed<br>Divorced	1<br>2<br>3<br>4	
42.	What is the age of your spouse? (if currently married and alive)	|___|___| Don't know	 98	
43.	What is his level of education?	No education<br>Literate but, no formal<br>education<br>Primary<br>Secondary<br>Higher secondary<br>Graduation<br>Above graduation<br>Don't know	1<br>2<br>3<br>4<br>5<br>6<br>7<br>8<br>98	
44.	Can you read and write?	Able to read only<br>Able to write only<br>Able to read and write<br>Cannot read or write	1<br>2<br>3<br>4	 <br><br>→ Skip to 47
45.	What is the highest level of education that you have completed?	No education<br>Primary<br>Secondary<br>Higher secondary<br>Graduation<br>Above graduation<br>Don't know	1<br>2<br>3<br>4<br>5<br>6<br>98	
46.	Years of schooling?	0 to 30 years<br>|___|___|		
47.	What is your current working status?	Working<br>Not Working<br>Retired<br>Never Worked	1<br>2<br>3<br>4	
48.	Occupation	Homemaker<br>Private<br>Public<br>Business<br>Agriculture<br>Home business<br>Daily wage labour<br>Hand craft from Home<br>Any Other (Specify)	1<br>2<br>3<br>4<br>5<br>6<br>7<br>8<br>98	
49.	Duration of work	Part time(less than 8 hr)<br>Full time (8 Hour)<br>All day(more than 8 hr)	1<br>2<br>3	
50.	Husband Working Status	Working<br>Not Working<br>Retired	1<br>2<br>3	

		Never Worked	4	
51.	Occupation of husband	Private<br>Public<br>Business<br>Agriculture<br>Home business<br>Daily wage labour<br>Hand craft from Home<br>Any Other (Specify)	1<br>2<br>3<br>4<br>5<br>6<br>7<br>98	
52.	Primary Source of income	From agriculture<br>From daily work<br>From govt. job<br>From private job<br>From shop/petty trade<br>Business<br>Others (specify)	1<br>2<br>3<br>4<br>5<br>6<br>98	
53.	Your monthly income (Average)	In Rupee		
54.	Do you read a newspaper or magazine?	Almost every day<br>At least once a week<br>Less than once a month<br>Not at all	1<br>2<br>3<br>4	
55.	Do you listen to the radio?	Almost every day<br>At least once a week<br>Less than once a month<br>Not at all	1<br>2<br>3<br>4	
56.	Do you watch television?	Almost every day<br>At least once a week<br>Less than once a month<br>Never	1<br>2<br>3<br>4	
57.	How many children do you have?			
58.	Of all the children, number of sons and daughters	Sons |____| Daughters |_____|		
59.	How many of your children are married?	Sons |____| Daughters |_____|		
60.	At what age each of your children got married and the age of their spouse at the time of marriage?<br>(eldest to youngest)	S. No. / Sex (M/F) / Age at marriage<br>1<br>2<br>3<br>4<br>5<br>6<br>7		

SECTION III: SOCIAL CAPITAL VARIABLES

Now, I would like to ask you some questions about your daughter and her social environment

S. No.	**General Questions**	Coding Categories		Skip/ Go to
61.	How much you know about Your daughter?	To a Great Extent<br>Somewhat<br>Very Little<br>Nothing	1<br>2<br>3<br>4	
62.	How much you love your daughter?	Don't love<br>Normal<br>Much<br>Very much	1<br>2<br>3<br>4	
63.	How much time do you spend with your daughter daily?	Less than an hour<br>One to two hour<br>More than two hour<br>All the time	1<br>2<br>3<br>4	
64.	How often you sit and talk with your daughter?	Daily<br>Once in a week<br>Two times in a week<br>Monthly	1<br>2<br>3<br>4	
65.	What kind of topic do you generally discuss with your daughter?	Family<br>Social<br>Educational<br>Health<br>Hobbies	1<br>2<br>3<br>4<br>5	
66.	Do you know about her hobbies?	Yes<br>No	1<br>2 →	Skip to 69
67.	What are her hobbies?	 Singing<br>Dancing<br>Painting and Crafts<br>Reading<br>Sports<br>Cooking<br>Writing<br>Other(specify)________<br>Nothing...........................	Yes No<br>1 2<br>1 2<br>1 2<br>1 2<br>1 2<br>1 2<br>1 2<br>96<br>98	
68.	Do you like and encourage her hobbies	Yes<br>No	1 →<br>2	Skip to 70
69.	If no , why?			
70.	Do you know about your daughter's ambitions?	Yes<br>No	1<br>2 →	Skip to 73
71.	What is her ambition? Or What she wants to become?	Doctor<br>Engineer<br>Teacher<br>Artist<br>Designer<br>Architect	1<br>2<br>3<br>4<br>5<br>6	

		Others specify<br>Nothing	7<br>8	
72.	Will you support her to fulfil it?	Yes<br>No	1<br>2	
73.	Does she share with you about her daily life problem	Yes<br>No	1 →<br>2	Skip to 75
74.	If no, If not what may be the reason according to you	Shyness<br>Communication gap<br>No close relation.<br>You don't have time to talk<br>Any other, specify.	1<br>2<br>3<br>4<br>98	
75.	If no, then with whom she would like to share?	Father<br>Siblings<br>Another family member<br>Friends<br>No one<br>Don't know	1<br>2<br>3<br>4<br>5<br>98	
76.	Does she ask for help from you for her any problem?	Yes<br>No	1<br>2 →	Skip to 79
77.	If yes, How you help her to deal with that problem?	Ask her to deal with own self<br>Guide and counselling her<br>Ask her to take help from other family members or friends<br>Discuss her problem with others<br>Did nothing	1<br>2<br>3<br>4<br>98	
78.	If you don't help her, what are the possible reasons?	Her problems are meaningless<br>She always have some problems<br>She is not that much important<br>I don't have time for her<br>I am not capable to solve<br>Other (specify)-----------------	1<br>2<br>3<br>4<br>8<br>98	

SECTION IV: Education

S. No.	Questions	Coding Categories		Skip/ Go to
79.	Do you think that education is important?	Yes<br>No	1<br>2	
80.	Do you think girls should be educated?	Yes<br>No	1<br>2 →	Skip to 83
81.	If yes, What level of education is important for girls?	Primary<br>Secondary<br>Higher<br>Other (Specify)	1<br>2<br>3<br>98	
82.	Do you want to educate your daughter?	Yes<br>No	1<br>2	
83.	If no , reason (specify)	 Girls should learn household work<br>Girls should get marry early<br>It will difficult to get a groom for her<br>She will be disobedient	Yes No<br>1 2<br>1 2<br>1 2<br>1 2<br>1 2	

		Family don't want to educate her<br>Our Community don't educate girls<br>She will not be beneficial for us<br>We have to save for her marriage<br>Others (Specify)________________<br>Don't know	1 2<br>1 2<br>1 2<br>96<br>98	
84.	Who takes the decision of your children's education in your home?	Children<br>Mother only<br>Mother with father<br>Father only<br>Father with other family members<br>Other family members only<br>Nobody	1<br>2<br>3<br>4<br>5<br>6<br>98	
85.	Do you talk about any education-related topics or discussion with your Children?	Yes<br>No	1<br>2	
86.	Did you face any trouble in educating your son?	Yes<br>No	1<br>2 →	Skip to 88
87.	If yes, what kind of problem you are facing?	 Financially<br>Family<br>Social<br>Health<br>Others<br>Don't know	Yes No<br>1 2<br>1 2<br>1 2<br>1 2<br>1 2<br>98	
88.	Did you face any trouble in educating your daughter?	Yes<br>No	1<br>2 →	Skip to 92
89.	If yes, what kind of problem you are facing?	 Financially.<br>Family<br>Social<br>Health<br>Others<br>Don't know	Yes No<br>1 2<br>1 2<br>1 2<br>1 2<br>1 2<br>98	
90.	What you usually do to deal with this problem?	Solving by own self<br>Talking to husband<br>Talking to family<br>Seeking help from outside of the family<br>Talking to school teacher<br>Nothing	1<br>2<br>3<br>4<br>5<br>98	
91.	Did you get any help from school regarding this?	Yes<br>No	1<br>2	
92.	Did your daughter face any problem in her studies	Yes<br>No	1<br>2 →	Skip to 94
93.	If yes, what kind of problems is that?	Financially<br>Family<br>Social<br>Health<br>Others<br>Don't know	1<br>2<br>3<br>4<br>5<br>98	
94.	Are you satisfied with her academic performance?	Yes<br>No	1<br>2	Skip to 97

95.	If no, what kind of obstacle is hindering her academic performance?	Yes No<br>Memory 1 2<br>Lack of interest 1 2<br>Lack of facilities 1 2<br>Health Issues 1 2<br>Family Pressure 1 2<br>Social Pressure 1 2<br>Other activities 1 2<br>Nothing 98	
96.	What you do to solve your daughter's study related problem	You teach her at home 1<br>Her father teach her 2<br>Other family members teach her 3<br>Other home tuition you provide 4<br>She went for Coaching classes 5<br>Severe punishment 6<br>Expert to handle (counselling) 7<br>Nothing 98	
97.	Do you want your daughter to go for higher studies?	Yes 1<br>No 2 →	Skip to 99
98.	Will you and your family support her?	Yes 1<br>No 2	
99.	If no, why?	____________________________<br>____________________________	
100	Will you allow your daughter to go outside of the city or state for higher study?	Yes 1<br>No 2 →	Skip to 102
101	Will your husband and family support you?	Yes 1<br>No 2	
102	If no, what will your next decision?	Will you try to convince them 1<br>You will go against their decision 2<br>You will be agreed with not to educate 3<br>your daughter 4<br>Nothing 98	
103	Does your family love your daughter?	Yes 1<br>No 2	
104	If no, Why?	______________________________<br>______________________________<br>______________________________	
105	Did you get any support from your family?	Emotional 1<br>Financial 2<br>Social 3	
106	How is your neighbourhood?	Good 1<br>Bad 2	
107	What kind of relation you share with your neighbourhood?	Friendly 1<br>Co-operative 2<br>Disputed 3<br>Violent 4	

		Don’t talk<br>Don’t no	5<br>98	
108	Do you feel your neighbourhood is safe for your daughter?	Yes<br>No	1<br>2	
109	Do you trust your neighbourhood?	Yes<br>No	1<br>2	
110	Will you share public gathering with your neighbourhood?	Yes<br>No	1<br>2	
111	Does your daughter has interaction with your neighbourhood?	Yes<br>No	1<br>2	
112	Does other girls of your neighbourhood are studying?	Yes<br>No	1<br>2	
113	If no, what could be the possible reasons?	Financial<br>Family problem<br>Social restriction<br>Early marriage<br>Health<br>Don’t know	1<br>2<br>3<br>4<br>5<br>98	
114	Did your daughter had face any type of critique or bullying from your neighbourhood?	Yes<br>No	1<br>2	
115	If yes, what you did to solve the matter?	 Seek help from family<br>Talk to the guilt person<br>Share this problem with community<br>Ask help from local authority<br>Report to administration of the area<br>Stop your girl to go outside of home<br>Did nothing	Yes No<br>1 2<br>1 2<br>1 2<br>1 2<br>1 2<br>1 2<br>98	

KIDSCREEN

**Physical Activities and Health (***Thinking about the last week from 104)*				
116	In general, how would your child rate her health?	Not at all<br>Slightly<br>Moderately<br>Very<br>Extremely	1<br>2<br>3<br>4<br>5	
117	Has your child felt physically fit and well?	Not at all<br>Slightly<br>Moderately<br>Very<br>Extremely	1<br>2<br>3<br>4<br>5	
118	Has your child been physically active (e.g.	Not at all<br>Slightly	1<br>2	

	running, climbing, biking)?	Moderately<br>Very<br>Extremely	3<br>4<br>5	
119	Has your child been able to run well?	Not at all<br>Slightly<br>Moderately<br>Very<br>Extremely	1<br>2<br>3<br>4<br>5	
120	Has your child felt full of energy?	Not at all<br>Slightly<br>Moderately<br>Very<br>Extremely	1<br>2<br>3<br>4<br>5	
**Feelings (***Thinking about the last week)*				
121	Has your child felt that life was enjoyable?	Not at all<br>Slightly<br>Moderately<br>Very<br>Extremely	1<br>2<br>3<br>4<br>5	
122	Has your child felt pleased that she is alive?	Not at all<br>Slightly<br>Moderately<br>Very<br>Extremely	1<br>2<br>3<br>4<br>5	
123	Has your child felt satisfied with her life?	Not at all<br>Slightly<br>Moderately<br>Very<br>Extremely	1<br>2<br>3<br>4<br>5	
124	Has your child been in a good mood?	Never<br>Almost Never<br>Sometimes<br>Almost always<br>Always	1<br>2<br>3<br>4<br>5	
125	Has your child felt cheerful?	Never<br>Almost Never<br>Sometimes<br>Almost always<br>Always	1<br>2<br>3<br>4<br>5	
126	Has your child had fun?	Never<br>Almost Never<br>Sometimes<br>Almost always<br>Always	1<br>2<br>3<br>4<br>5	
**General Mood (***Thinking about the last week)*				
127	Has your child felt that she does everything badly?	Never<br>Almost Never<br>Sometimes<br>Almost always<br>Always	1<br>2<br>3<br>4<br>5	
128	Has your child felt sad?	Never<br>Almost Never<br>Sometimes	1<br>2<br>3	

		Almost always<br>Always	4<br>5	
129	Has your child felt so bad that she didn't want to do anything?	Never<br>Almost Never<br>Sometimes<br>Almost always<br>Always	1<br>2<br>3<br>4<br>5	
130	Has your child felt that everything in her life goes wrong?	Never<br>Almost Never<br>Sometimes<br>Almost always<br>Always	1<br>2<br>3<br>4<br>5	
131	Has your child felt fed up?	Never<br>Almost Never<br>Sometimes<br>Almost always<br>Always	1<br>2<br>3<br>4<br>5	
132	Has your child felt lonely?	Never<br>Almost Never<br>Sometimes<br>Almost always<br>Always	1<br>2<br>3<br>4<br>5	
133	Has your child felt under pressure?	Never<br>Almost never<br>Sometimes<br>Almost always<br>Always	1<br>2<br>3<br>4<br>5	
**About Your Child (***Thinking about the last week)*				
134	Has your child been happy with the way she is?	Never<br>Almost never<br>Sometimes<br>Almost always<br>Always	1<br>2<br>3<br>4<br>5	
135	Has your child been happy with her clothes?	Never<br>Almost never<br>Sometimes<br>Almost always<br>Always	1<br>2<br>3<br>4<br>5	
136	Has your child been worried about the way she looks?	Never<br>Almost never<br>Sometimes<br>Almost always<br>Always	1<br>2<br>3<br>4<br>5	
137	Has your child felt jealous of the way other girls look	Never<br>Almost never<br>Sometimes<br>Almost always<br>Always	1<br>2<br>3<br>4<br>5	
138	Has your child wanted to change something about her body?	Never<br>Almost never<br>Sometimes<br>Almost always<br>Always	1<br>2<br>3<br>4<br>5	

**Free Time (***Thinking about the last week)*				
139	Has your child had enough time for herself?	Never<br>Almost never<br>Sometimes<br>Almost always<br>Always	1<br>2<br>3<br>4<br>5	
140	Has your child been able to do the things that she wants to do in her free time?	Never<br>Almost never<br>Sometimes<br>Almost always<br>Always	1<br>2<br>3<br>4<br>5	
141	Has your child had enough opportunity to be outside?	Never<br>Almost never<br>Sometimes<br>Almost always<br>Always	1<br>2<br>3<br>4<br>5	
142	Has your child had enough time to meet friends?	Never<br>Almost never<br>Sometimes<br>Almost always<br>Always	1<br>2<br>3<br>4<br>5	
143	Has your child been able to choose what to do in her free time?	Never<br>Almost never<br>Sometimes<br>Almost always<br>Always	1<br>2<br>3<br>4<br>5	
**Family and Home Life** (*Thinking about the last week)*				
144	Has your child felt understood by her parent(s)?	Not at all<br>Slightly<br>Moderately<br>Very<br>Extremely	1<br>2<br>3<br>4<br>5	
145	Has your child felt loved by her parent(s)?	Not at all<br>Slightly<br>Moderately<br>Very<br>Extremely	1<br>2<br>3<br>4<br>5	
146	Has your child been happy at home?	Never<br>Almost never<br>Sometimes<br>Almost always<br>Always	1<br>2<br>3<br>4<br>5	
147	Has your child felt that her parent(s) had enough time for her?	Never<br>Almost never<br>Sometimes<br>Almost always<br>Always	1<br>2<br>3<br>4<br>5	
148	Has your child felt that her parent(s) treated her fairly?	Never<br>Almost never<br>Sometimes<br>Almost always<br>Always	1<br>2<br>3<br>4<br>5	
149	Has your child been able	Never	1	

	to talk to her parent(s) when he/she wanted to?	Almost never<br>Sometimes<br>Almost always<br>Always	2<br>3<br>4<br>5	
**Money matters** (*Thinking about the last week)*				
150	Has your child had enough money to do the same things as her friends?	Never<br>Almost never<br>Sometimes<br>Almost always<br>Always	1<br>2<br>3<br>4<br>5	
151	Has your child felt that she had enough money for his/her expenses?	Never<br>Almost never<br>Sometimes<br>Almost always<br>Always	1<br>2<br>3<br>4<br>5	
152	Does your child feel that she has enough money to do things with her friends?	Not at all<br>Slightly<br>Moderately<br>Very<br>Extremely	1<br>2<br>3<br>4<br>5	
**Friends** (*Thinking about the last week)*				
153	Has your child spent time with her Friends?	Never<br>Almost never<br>Sometimes<br>Almost always<br>Always	1<br>2<br>3<br>4<br>5	
154	Has your child done things with other girls and boys?	Never<br>Almost never<br>Sometimes<br>Almost always<br>Always	1<br>2<br>3<br>4<br>5	
155	Has your child had fun with her friends?	Never<br>Almost never<br>Sometimes<br>Almost always<br>Always	1<br>2<br>3<br>4<br>5	
156	Have your child and /her friends helped each other?	Never<br>Almost never<br>Sometimes<br>Almost always<br>Always	1<br>2<br>3<br>4<br>5	
157	Has your child been able to talk about everything with her friends?	Never<br>Almost never<br>Sometimes<br>Almost always<br>Always	1<br>2<br>3<br>4<br>5	
158	Has your child been able to rely on her friends?	Never<br>Almost never<br>Sometimes<br>Almost always<br>Always	1<br>2<br>3<br>4<br>5	

School and Learning (*Thinking about the last week)*

159	Has your child been happy at school?	Not at all<br>Slightly<br>Moderately<br>Very<br>Extremely	1<br>2<br>3<br>4<br>5	
160	Has your child got on well at school?	Not at all<br>Slightly<br>Moderately<br>Very<br>Extremely	1<br>2<br>3<br>4<br>5	
161	Has your child been satisfied with her teachers?	Not at all<br>Slightly<br>Moderately<br>Very<br>Extremely	1<br>2<br>3<br>4<br>5	
162	Has your child been able to pay attention?	Never<br>Almost never<br>Sometimes<br>Almost always<br>Always	1<br>2<br>3<br>4<br>5	
163	Has your child enjoyed going to school?	Never<br>Almost never<br>Sometimes<br>Almost always<br>Always	1<br>2<br>3<br>4<br>5	
164	Has your child got along well with her teachers?	Never<br>Almost never<br>Sometimes<br>Almost always<br>Always	1<br>2<br>3<br>4<br>5	
**Bullying** (*Thinking about the last week)*				
165	Has your child been afraid of other girls and boys?	Never<br>Almost never<br>Sometimes<br>Almost always<br>Always	1<br>2<br>3<br>4<br>5	
166	Have other girls and boys made fun ofyour child?	Never<br>Almost never<br>Sometimes<br>Almost always<br>Always	1<br>2<br>3<br>4<br>5	
167	Have other girls and boys made fun ofyour child?	Never<br>Almost never<br>Sometimes<br>Almost always<br>Always	1<br>2<br>3<br>4<br>5	
